# Cut throat injuries at a university teaching hospital in northwestern Tanzania: a review of 98 cases

**DOI:** 10.1186/1471-227X-14-1

**Published:** 2014-01-14

**Authors:** Japhet M Gilyoma, Kiyeti A Hauli, Phillipo L Chalya

**Affiliations:** 1 Otorhinolaryngology unit, Bugando Medical Centre, Mwanza, Tanzania; 2 Department of Surgery, Catholic University of Health and Allied Sciences-Bugando, Mwanza, Tanzania; 3 Department of Psychiatry, Catholic University of Health and Allied Sciences-Bugando, Mwanza, Tanzania

**Keywords:** Cut throat injuries, Etiology, Patterns, Treatment outcome, Tanzania

## Abstract

**Background:**

Cut throat injuries though rarely reported in literature pose a great therapeutic challenge because multiple vital structures are vulnerable to injuries in the small, confined unprotected area. A sudden increase in the number of cut throat patients in our centre in recent years prompted the authors to analyze this problem. This study was conducted in our local setting to describe the etiology, patterns and treatment outcome of these injuries.

**Methods:**

This was a combined retrospective and prospective study of cut throat injury patients who were managed at Bugando Medical Centre between February 2009 and January 2013. Statistical data analysis was done using SPSS software version 17.0.

**Results:**

A total of 98 patients with cut throat injuries were studied. Males outnumbered females by a ratio of 2.4: 1. The median age of patients was 26 years (range 8 to 78 years). Majority of patients (79.6%) had no employment and most of them (65.3%) came from rural community. Homicide was the commonest (55.1%) cause, followed by suicidal attempts (34.7%) and accidental (10.2%) injuries. Interpersonal conflict (24.4%) was the most common motivating factor for homicidal injury whereas psychiatric illness (16.2%) and road traffic accidents (9.2%) were the most frequent motivating factors of suicidal attempt and accidental injuries respectively. The majority of injuries were in Zone II accounting for 65.3% of cases and most of them had laryngeal (57.1%) injury. Surgical debridement, laryngeal/hypopharynx repair and tracheostomy were the most common surgical procedures performed in 93.9%, 73.5% and 70.4% of patients respectively. Postoperative complication rate was 57.1%, the commonest being surgical site infections in 28.1% of patients and it was significantly associated with late presentation and anatomical zones (P < 0.001). The overall median duration of hospitalization was 12 days. Patients who had postoperative complications stayed longer in the hospital and this was statistically significant (*p* = 0.011). Mortality rate was 11.2% and was significantly associated with co-morbidities, delayed presentation and presence of complications (*p* < 0.001). The follow up of patients was poor.

**Conclusions:**

Cut throat injuries are a major cause of morbidity and mortality among young adult males in our setting. Addressing the root causes of violence such as poverty, unemployment, and substance abuse will reduce the incidence of these injuries in our environment.

## Background

Cut throat injuries are a unique form of trauma that is potentially devastating and associated with substantial emotional, physical and financial burden on community and hospital resources [1]. Cut throat injuries causes profound morbidity due to prolonged hospitalization, high cost of health care, loss of productivity and reduced quality of life and above all death [1,2].

Globally, cut throat injuries account for approximately 5% to 10% of all traumatic injuries with multiple structures being injured in 30% of patients [3-7]. However, in developing countries the incidence is increasing at a fast rate partly because of increasing conflict over limited resources, poor socioeconomic status, poverty, unemployment, easy access to firearms, alcohol and substance misuse and increased crime rates [8].

The etiology of cut throat injuries can be broadly divided into suicidal, homicidal or accidental in occurrence [3,9]. Familial troubles, psychiatric illnesses and poverty are documented triggering factors in suicidal attempts. The triggering factors for homicide are political conflict, familial, land related disputes and sex related crimes [9-11]. Regarding accidental causes mostly related to the road traffic accident and fall injuries [10].

Cut throat injuries pose a great challenge because multiple vital structures are vulnerable to injuries in the small, confined unprotected area [9]. Up to 30% of the injuries involve multiple structures [4-7]. The management of these injuries requires a multidisciplinary approach requiring the close collaboration of the Otolaryngologist, the anesthetist and the psychiatrist [11-15]. The anesthetist secures an uncompromised airway and makes sure the patient is breathing; the otolaryngologist assesses the injury and repairs the severed tissues with the aim of restoration of swallowing, phonation and breathing. The psychiatrist provides adequate care and supervision during and after surgical treatment [9,11,14,15]. However, in most developing countries such as Tanzania, late presentation to health facilities coupled with lack of advanced pre-hospital and ineffective ambulance system for transportation of patients to hospital care contributes significantly to increasing morbidity and mortality [9,16,17].

There is paucity of information in most developing countries including Tanzania on cut throat injuries where greater emphasis has been placed on injuries related to Road traffic accidents, which are more common [9-11]. A sudden increase in the number of admissions of patients with cut throat injuries in our setting prompted the authors to analyze this problem. This study was conducted in our local setting to describe our own experience in the management of cut throat injuries, outlining the etiology, patterns and treatment outcome of these injuries with the hope that our findings will be a guide to offer preventive and therapeutic measures in these patients and ultimately improve their outcome.

## Methods

This was a combined retrospective and prospective study of cut throat injury patients who presented to the Accident and Emergency of Bugando Medical Centre (BMC) between February 2009 and January 2013. BMC is a referral, consultant and teaching hospital for the Catholic University of Health and Allied Sciences-Bugando (CUHAS-Bugando) and other paramedics and it is located in Mwanza city in the northwestern part of the United Republic of Tanzania. It is situated along the shore of Lake Victoria and has 1000 beds. BMC is one of the four largest referral hospitals in the country and serves as a referral centre for tertiary specialist care for a catchment population of approximately 13 million people from neighboring regions in northwestern Tanzania. There is no trauma centre or established advanced pre-hospital care in Mwanza city as a result all trauma patients are referred to BMC for expertise management. All patients who presented with cut throat injury during the study period were included in the study. Patients who presented in a “shocked” state and those who were under 18 years of age, their parents, guardian or relatives had to consent on their behalf. Patients with incomplete data and those who were brought in dead were excluded from the study. Minor neck injury not required admission and patient with minor trauma in the neck but major trauma in other parts of the body need hospitalization were excluded from the study. The details of patients who presented from February 2009 to September 2010 were retrieved retrospectively from patient registers kept in the Medical record departments, the surgical wards, and operating theatre. Patients who presented to the A & E department between October 2010 and January 2013 were prospectively enrolled in the study after signing an informed written consent for the study.

All recruited patients were, before enrolled in the study, resuscitated in the A&E department according to Advanced Trauma Life Support (ATLS). From the A & E department, patients were taken to theatre for surgical intervention and from there; patients were taken into the Otorhinolaryngology wards or the intensive care unit (ICU) for admission.

All the data regarding study population were collected and compiled in a structured questionnaire with thoroughly looked upon ethical implication. All the data pertinent to the patient kept confidential. Data were categorized according to the demographic pattern of the patient, cause and motivating factors behind the injury, prehospital care, site of the neck injury (according to the defined zone of the neck), type and extend of the tissue damage or involved, presentation during admission, time taken or delay from the incidence to the hospital attendance (injury-arrival interval) and duration of the hospital stay, type of special intervention given, records of mortality, noticeable morbidity and outcome. Patients who were prospectively enrolled in the study were followed up till discharge or death and thereafter for up to 12 months after surgery.

### Statistical data analysis

Statistical data analysis was done using SPSS software (Statistical Package for the Social Sciences, version 17.0, SPSS Inc, Chicago, Ill, USA). Data was summarized in form of proportions and frequent tables for categorical variables. Continuous variables were summarized using means, median, mode and standard deviation. p-values were computed for categorical variables using Chi-square (*χ*2) test and Fisher’s exact test depending on the size of the data set. Independent student *t*-test was used for continuous variables. Multivariate logistic regression analysis was used to determine predictor variables that are associated with outcome. A p-value of less than 0.05 was considered to constitute a statistically significant difference.

### Ethical considerations

The study was carried out after the approval by the department of surgery and BMC/CUHAS-Bugando ethics review board. An informed written consent was sought from patients/relatives who were recruited prospectively.

## Results

**Figure 1:**
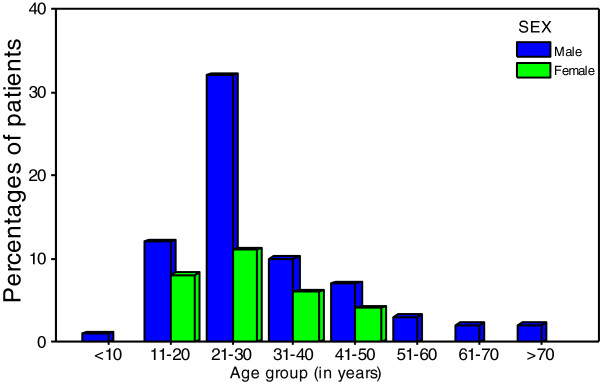
Distribution of age group according to sex.

During the period of study, a total of 114 patients presented to our centre with cut throat injuries. Out of these, 16 patients were excluded from the study due to failure to meet the inclusion criteria and incomplete data. Thus, 98 patients were enrolled into the study. Of these, 12 (12.2%) patients were studied retrospectively and the remaining 86(87.8%) patients were studied prospectively. There were 69 (70.4%) males and 29 (29.6%) females with a male to female ratio of 2.4: 1. The age of victims ranged from 8 to 78 years with a median age of 26 years. The peak age incidence was in the age group of 21-30 years and accounted for 43.9% of cases (Figure 1). The vast majority of patients, 74 (75.5%) had primary or no formal education and most of them (78, 79.6%) had no employment. Sixty-four (65.3%) patients came from rural areas around Mwanza City. The majority of patients, 92 (93.9%) were belong to the low socioeconomic class and only 6 (6.1%) victims were from higher classes. Only seven (7.1%) of the victims had definable source of private or governmental health care insurance at the time of their injury.

Regarding the causes and motivating factors for cut throat injury, fifty-four (55.1%) patients were due to homicidal injury, 34(34.7%) victims were due to suicidal attempt and only 10(10.2%) person were due to accidental injury. Interpersonal conflict ( 24.4%) was the most common motivating factor for homicidal injury whereas psychiatric illness (16.2%) and road traffic accidents (9.2%) were the most frequent motivating factors of suicidal attempt and accidental injuries respectively (Table 1). Associated medical co-morbidities were reported in 22 (22.4%) patients. these included; psychiatric illness in 16 (72.7%) patients and diabetes mellitus, hypertension and chronic chest infection in two (9.1%) patients each respectively.

**Table 1:** Distribution of patients according to the cause and motivating factors of cut throat injury (N = 98)

**Cause of the cut throat injury**	**Motivating factors of cut throat injury**	**Frequency**	**Percentage**
**Homicidal injury**	Interpersonal conflict	24	24.5
	Land dispute	12	12.2
	Family problem	8	8.2
	Robbery	4	4.1
	Sexual violence	2	2.0
	Political conflict	2	2.0
	Others	2	2.0
	**Total**	**54**	**55.1**
**Suicidal attempt**	Psychiatric illness	16	16.3
	Familial disharmony	10	10.2
	Sexual violence/abuses	3	3.1
	Loss of employment	3	2.0
	Substance abuse	2	2.0
	**Total**	**34**	**34.7**
**Accidental /unintentional injury**	Road traffic accidents	9	9.2
	Fall from a height	1	1.0
	**Total**	**10**	**10.2**

The majority of injuries were in Zone II accounting for 65.3% of cases and most of them had laryngeal (57.1%) injury as shown in Table 2. During presentation, the majority of victims presented with open wounds (93.9%) and active bleeding (82.6%). Preoperative hemorrhagic shock and respiratory distress was recorded in 22.4% and 16.3% of cases respectively. The vast majority of patients, 68(69.4%) reported to the A & E department within 24 hours after injury. None of the patients received any pre-hospital care and majority of them (76, 77.6%) were brought in by relatives, friends or Good Samaritan, 16(16.3%) by police and only 6 (6.2%) patients were brought in by ambulance (Table 2). The waiting time (i.e. time interval taken from reception at the A & E department and reception of treatment) ranged from 30 minutes to ten hours with a median of 4 hours. The majority of patients, 75 (76.5%) were attended to within 2-4 hours of arrival to the A & E department. All patients in this study underwent surgical procedures as depicted in Table 3. Surgical debridement, laryngeal/hypopharynx repair and tracheostomy were the most common surgical procedures performed accounting for 93.9%, 73.5% and 70.4% of patients respectively. Blood transfusion was recorded in 45.9% of cases.

**Table 2:** Anatomical site, structures injured, presentation and injury-arrival time

**Variables**	**Response**	**Frequency**	**Percentage**
**Anatomical site**	Zone I	14	14.3
	Zone II	64	65.3
	Zone III	10	10.2
**Structures injured**	Skin, platysma & fascia (superficial & deep)	98	100
	Larynx	56	57.1
	Hypopharynx	34	34.7
	Thyroid gland and cartilage	23	23.5
	Trachea	14	14.3
	Internal jugular vein	8	8.2
	Carotid vessels	3	3.1
**Presentation**	Open wounds	92	93.9
	Active bleeding	80	82.6
	Applied bandage on the wound	74	75.5
	Inadequate wound management	45	45.9
	Proper wound management	34	34.7
	Hemorrhagic shock	22	22.4
	Respiratory distress	16	16.3
**Injury-arrival time**	< 6 hours	6	6.1
	6-12 hours	14	13.3
	12-24 hours	48	49.0
	>24 hours	30	30.6

**Table 3:** Distribution of patients according to the type of treatment/surgical procedure provided

**Type of treatment/surgical procedure provided**	**Frequency**	**Percentage**
Surgical debridement	92	93.9
Laryngeal & hypopharynx repair	72	73.5
Tracheostomy	69	70.4
Simple repair & closure	56	57.1
Ligation of major veins	8	8.2
Ligation of major arteries	3	3.1
Blood transfusion	45	45.9

Fifty-six (57.1%) patients developed sixty-four complications of which surgical site infections (28.1%) was the most common complications (Table 4). Complication rate was significantly associated with delayed presentation and anatomical zones (*p* < 0.001).

**Table 4:** Distribution of patients according to postoperative complications (N = 64)

**Postoperative complications**	**Frequency**	**Percentages**
Surgical site infection	18	28.1
Persistence voice change	14	21.9
Hemorrhagic shock	8	12.5
Respiratory distress	6	9.4
Aspiration pneumonia	5	7.8
Ugly scar	4	6.3
Laryngeal stenosis	3	4.7
Pharyngeal stenosis	2	3.1
Permanent tracheostomy	2	3.1
**Total**	**64**	**100**

The overall length of hospital stay (LOS) ranged from 1 to 72 days with a median of 12 days. The median LOS for non-survivors was 5 days (range 1-12 days). The majority of patients, 69 (70.4%) stayed in hospital less than two week duration. Patients who had post complications stayed longer in the hospital and this was statistically significant (P = 0.011).

In this study, eleven patients died giving a mortality rate of 11.2%. According to multivariate logistic regression analysis, associated co-morbidities (OR = 1.6, 95% C.I. (1.2- 4.8), P = 0.011), delayed presentation (OR = 8.4, 95% CI (6.6- 16.4), *p* = 0.020) and presence of complications (OR = 11.8, 95% CI (10.1-14.5), p = 0.001) were the main predictors of mortality.

Of the survivors, 78 (89.7%) patients were discharged well, 6 (6.8%) patients were discharged against medical advice and the remaining 3 (3.4%) patients were discharged with permanent disabilities related to permanent tracheostomy and permanent voice change. Of the survivors, only 32 (36.8%) patients were available for follow-up at 6–12 months and the remaining 55 (63.2%) patients were lost to follow-up.

## Discussion

In this review, most of cut throat injury patients were young in their third decade of life and tended to affect more males than females, a finding which agrees with findings reported elsewhere [3,9,11]. Male preponderance in this age group is attributable to their active participation in risk taking behaviors and their frequent involvement in interpersonal violence. This has great economic impact since these are people in their most productive years and the injuries impose a considerable burden on their families and the society as a whole.

In agreement with other studies ( 3, 9, 11), most of patients in this study were unemployed and uneducated and the majority of them came from low-income areas of the city and only few had definable source of health care insurance at the time of their injury. This observation has an implication on accessibility to health care facilities. Unemployment can act as a stressful life event leading to suicide [18] with studies suggesting an increase in the parasuicide and suicide rates among unemployed individuals than in the general population [19]. Socioeconomic improvement of otherwise normal individuals by provision of jobs for example and family planning education can eliminate the triggering factor of unemployment.

Regarding the causes of cut throat injuries, the majority of patients in this study were due to homicidal injury and the remaining patients were due to suicidal attempt and accidental injury. Interpersonal conflict was the most common motivating factor for homicidal injury whereas psychiatric illness and road traffic accidents were the most frequent motivating factors of suicidal attempt and accidental injuries respectively. Similar finding was also reported in Bangladesh by Manilal et al. [9]. On the contrary, cut throat was reported to be suicidal in majority of cases in western studies [20,21].

In this study, associated medical co-morbidities were reported in 22.4% of cases. Of these, psychiatric illness accounted for more than seventy percent of cases. This observation agrees with other studies done elsewhere [3,9,11]. As found in our study, psychiatric illness has been reported in literature to be associated with suicidal attempt [22,23]. Psychiatric illnesses are the strongest predictors of suicide [23]. Suicide occurs 20.4 times more frequently in individuals with psychiatric illness than the general population [22-24].

The prehospital care of trauma patient has been reported to be the most important factor in determining the ultimate outcome after the injury [25]. None of our patients had pre-hospital care; as a result the majority of them were brought in by relatives, Good Samaritan and police who are not trained on how to take care these patients during transportation. Only 6.2% of patients in this study were brought in by ambulance. This observation is common to many other developing countries [25,26]. The lack of advanced pre-hospital care and ineffective ambulance system for transportation of patients to hospitals are a major challenges in providing care for trauma patients in our environment and have contributed significantly to poor outcome of these patients.

The majority of injuries in this study were in Zone II and most of them had laryngeal injury which is in keeping with other studies [9-11]. Zone ll injuries are those occurring in the region between the cricoid cartilage and the angle of the mandible. The predominance of zone II injuries in this study may be attributable to the fact that unlike zones l and lll, zone II is not protected by bony structures making it more vulnerable to injuries. Injuries in this zone are the easiest to expose and evaluate [9-13].

As reported by others [3,9], majority of patients in this study presented with open wounds and active bleeding. Hemorrhagic shock and respiratory distress were reported in only 22.4% and 16.3% of cases. Exposed hypopharynx and or larynx following cut throat, hemorrhage, shock and asphyxia from aspirated blood are commonest cause of death following cut throat injury. A good knowledge of the nature and type of cut throat wounds allows the clinicians to understand the type weapon used and this is of great importance for medico-legal purposes and surgical treatment.

In this study, surgical debridement, laryngeal/hypopharynx repair and tracheostomy were the most common surgical procedures performed. Similar treatment patterns were reported by other authors [3,9-11]. Cut throat injuries require a multidisciplinary approach involving the anesthetist and psychiatrists working in conjunction with the Otolaryngologist and could be managed with better prognosis if the patients present early to the hospital and are given prompt attention [11,14,15]. In this study, all patients that attempted suicide were considered for the psychiatric consultation. This was because the act of suicide is a sign of underlying mental illness and there is possibility of a second attempt [9,22].

The presence of complications has an impact on the final outcome of patients presenting with cut throat injuries as supported by the present study. In keeping with other studies [3,9,11], more than fifty percent of patients developed complications of which surgical site infections was the most common complications. Complication rate was significantly associated with delayed presentation and anatomical zones. Early recognition and management of complications following cut throat injury is of paramount in reducing the morbidity and mortality resulting from these injuries.

Prevention of these complications depends upon immediate resuscitation by securing the airway by tracheostomy or intubation, prompt control of external hemorrhage and blood replacement, protection of the head and neck, accurate and rapid diagnosis, and prompt intervention or operative treatment when indicated.

The length of hospital stay has been reported to be an important measure of morbidity among trauma patients. Prolonged hospitalization is associated with an unacceptable burden on resources for health and undermines the productive capacity of the population through time lost during hospitalization and disability [1,2,9]. The median duration of hospital stay in this study was found to be longer than that reported by other authors [3,9]. This can be explained by large number of patients with postoperative complications which usually need long duration of hospitalization.

The present study had a mortality rate of 11.2%, which is higher than the rate quoted by Manilal et al. [9]. Factors responsible for the high mortality rate in our study were associated co-morbidities, delayed presentation and presence of complications.

In our study, the cut throat injuries were successfully without complications in 89.7% of cases which is similar to other studies reported elsewhere [9,11].

Self discharge by patient against medical advice is recognized problem in our setting and this is rampant, especially amongst surgical patients. Similarly, poor follow up visits after discharge from hospitals remain a cause for concern. In the present study, only 36.8% of survivors were available for follow up which is in keeping with other studies done in developing countries [3,9,11].

The potential limitation of this study is the fact that information about some patients was incomplete in view of the retrospective nature of the study. This might have introduced some bias in our findings.

## Conclusions

Cut throat injuries have become a major cause of morbidity and mortality among young males in our society where resources for prehospital and hospital trauma care are limited. High rates of unemployment, poor socio-economic status, poor education, poverty and substance abuse have been reported to be responsible for these injuries in our society. Addressing the root causes of violence such as poverty, unemployment, and substance abuse will reduce the incidence of cut throat injuries in our environment. Establishment of efficient emergency health care services for pre-hospital care and effective ambulance system for rapid transport of injured victims to hospital will reduce morbidity and mortality associated with these injuries.

## Competing interests

The authors declare that they have no competing interests.

## Authors’ contributions

JMG conceived the study, participated in the design and coordination of the study and drafted the manuscript. KAH and PLC contributed in study design, literature search, data analysis, manuscript writing and editing. In addition PLC submitted the manuscript. All the authors read and approved the final manuscript.

## Pre-publication history

The pre-publication history for this paper can be accessed here:

http://www.biomedcentral.com/1471-227X/14/1/prepub
